# Research barriers from experts’ viewpoints who attended the research workshops of Mazandaran University of Medical Sciences

**Published:** 2015

**Authors:** M Ataee, A Hesamzadeh, M Kheradmand

**Affiliations:** * Nursing Department, Deputy of Research and Technology, Mazandaran University of Medical Sciences, Sari, Iran; ** Community Medicine, Deputy of Research and Technology, Mazandaran University of Medical Sciences, Sari, Iran

**Keywords:** research barriers, research workshop, factor analysis

## Abstract

**Introduction.** Experts who work in the medical center could play a significant role in doing research directed to the prevention and treatment of the diseases. The aim of the present study was to identify personal and organizational barriers in order to do the research expressed by the experts who participated in the workshop.

**Methods.** This study was a descriptive research, in which the viewpoints of 250 experts who attended an educational seminar on research from 2007 to 2009 were selected by the census. To collect the data, a valid and reliable questionnaire, in Likert scale, consisting of 3 parts, individual characteristics, personal and organizational barriers were applied. The data report was done by using the T-test and the analysis of variance. An exploratory factor analysis was also conducted to summarize the data and to classify the questionnaire variables.

**Results.** From the 250 distributed questionnaires, 213 (85.2%) were returned. The most important personal barriers of research were spare time and lots of work. The most important organizational barrier was the lack of motivation from the managers. The factor analysis revealed a lack of knowledge and information, inadequate time and lots of work and a lack of incentive, as a personal barrier, and inadequate organizational support, inadequate consultant and library services, insufficient facilities for sampling, poor access to the samples and methods of doing research, not enough cooperation of the colleagues, poor facilities and counseling were identified as organizational barriers.

**Conclusion.** Recognizing personal and regulatory barriers in research for the working experts who attended the educational workshop of research methodology could help the managers and the officials remove these obstacles and in turn use the manpower of the university sufficiently.

## Introduction

Research (scientific and systematic review) is one of the fundamental pillars of the development of human society. In addition, any movement of scientific and reasonable terms is not possible without the support of research. In fact, the study is called as one of the important indicators of growth [**1**-**3**]. The institution of higher education as a manufacturing and dissemination of knowledge has a vital role in the growth and sustainable development of the country. Training of human resources, development, and growth of knowledge, identifying problems and doing research on them are the main tasks of the institution [**4**].

In developing countries, developing the capacity is one of the strongest, most cost-effective and lasting development tools in the health and development [**5**,**6**]. Health research in the field of health systems is to provide better health care, which is more equitable and less discriminative [**7**]. However, the research conducted in the developing countries is not desirable and, compared to the developed countries, human resources, budget and facilities spent on research are trivial [**4**]. Especially in the last decade, research has been engaged in studying its barriers from the viewpoint of faculty members. These reflect the fact that there are always many obstacles, including the lack of appropriate budget allocations, timely and accurate information, the rapid changes in management and laws, the lack of professional researchers, poor selection, and administration, the lack of hindering research program, that block the pathway of research [**4**,**5**,**8**]. However, this study was conducted from the views of the experts working at the University of Medical Sciences in the research on obstacles. A study that was performed on nurses working in hospitals of Shahrekord showed that the most substantial barriers of research were lack of time and being busy, although family responsibilities were not enough points [**5**]. Based on the Public Relations Department of the Ministry of Health and Medical Education, there are about 97,198 people employed in the department (35% of the total employees) and those with a bachelor’s, master’s degree and doctorate [**9**], who, regarding to Islamic Republic of Iran Vision 2025, have been emphasized to achieve first place in the economy, science and technology in the region of Southwest Asia [**10**], an could play a significant role in achieving this goal. According to the comprehensive plan of the health and Horizon 2025 document, that requires universities to reach the degree of knowledge production, so that the area could be proposed as a scientific first pole. The task of schools is more sensitive in this regard and in improving training and the increase of the number of research projects there is a need to mobilize everyone, experts, and faculty members to conduct research projects.

In this regard, Mazandaran University of Medical Sciences held training workshops for experts while working in university research from previous years. However, there were an impressive number of participants in the workshops of the research projects presented by the experts. In general, the barriers of doing research can be divided into two categories: institutional barriers and individual barriers [**11**]. Little investigation has been conducted to identify the individual and organizational barriers, and this study tried to examine the factors that determine individual and regulatory obstacles of research from the viewpoint of the workshop participants in Mazandaran University of Medical Sciences 2007-2009. 

**Methodology**

This study was a cross-sectional one to verify the views of the experts participating in the seminar method held by the Department of Science and Technology of Mazandaran University of Medical Sciences in the years 2007 to 2009. The study population included 250 experts who participated in the training workshops research method and were employed in the health centers of Mazandaran University of Medical Sciences, being selected by census method. Criteria for the study was used in offices and health centers affiliated to Tehran University of Medical Sciences and traversed at least six months from the date of the workshop.

The data collection questionnaire consisted of two parts: 1. Individual characteristics of the subjects and 2. Individual and organizational barriers. The severity of problems based on the 5 points Likert scale was graded from low to high (1 = completely disagree, 2 = disagree, 3 = 4 = agree, 5 = completely agree with the idea). This questionnaire was used to investigate the nature and colleagues in 2007, and its validity and reliability was confirmed (r=0.84) [**3**]. In order to reaffirm the validity of the questionnaire, the researcher provided information to experts and faculty members, that the ultimate validity was confirmed. Cronbach’s alpha factor was used to determine the reliability of the instrument after the questionnaires were distributed among 38 participants and the equivalent workshop ratio was 81.0. Questionnaires were distributed by mail or in person between the experts participating in the seminar. The information was then entered in the computer by using descriptive statistics such as average, standard deviation, etc. The exploratory factor analysis was recognized as a method of multivariate analysis together with the aim of obtaining the pattern prevailing obstacles in the conduction of the research. Moreover, inferential statistics, such as correlation, was analyzed with SPSS 17.0 software.

**Findings**

A total of 250 questionnaires were distributed and 213 (85.2 percent response rate) were returned. 40.4 percent of the total cases (86) were males. The average age of the respondents was 37.10 ± 5.98 years. Other information regarding the participants was demonstrated in **Table 1**. 

**Table 1:** Demographic characteristics of participants

Variable	number (percentage)
Gender	Men: 86 (40.4%)
	Women: 127 (59.6%)
Academic degree	Bachelor: 153 (71.8%)
	Masters and above: 60 (28.2%)
Occupation	Medic: 47 (22.1%)
	Nurse: 42 (19.7%)
	Obstetrician: 23 (10.8%)
	Health expert: 52 (24.4%)
	Other: 49 (23.0%)
Employment Status	Official: 132 (62.0%)
	Projective: 4 (2.3%)
	Contractual: 64 (30.5%)
	Corporative: 11 (5.2%)
Service location	Hospitals: 58 (24.4%)
	Health centers: 128 (60.1%)
	Staff offices: 33 (15.5%)

44.6 of the participants reported that they had a history of research activities. 57.3 percent stated that the investigation activities were not conducted in the field of their work, or they did not know about it. According to the 61.0% of the subjects, the authorities did not ask them to do research work. 47.4% of these people were unaware of the research priorities in their field.

The results showed an individual barriers minimum score of 21 and a maximum of 102, with an average of 47.09 ± 9.50. In this regard, the main obstacles were the lack of time and individual business research, being apart from academic centers and lack of motivation. Unfamiliarity with the statistical principles was mentioned as another significant obstacle research and the least important barriers were no benefits for the patient or the clients and the inability to use a computer. 

The organizational barriers minimum score was of 34.0 and the maximum of 99.0, with an average of 73.39 ± 10.20 respectively. The most significant regulatory barriers mentioned were research conducted by researchers from the authorities, lack of motivation, lack of research, and lack of an adequate funding for the research of the consultation. The least important barriers were ethical limits research and lack of cooperation of health centers with the authorities.

The organization factor analysis was used to determine the factors influencing research in both the personal barriers and the obstacles prevention. Based on the findings in the personal barriers, the KMO (Kaiser-Meyer-Olkin Measure of Sampling Adequacy KMO) amount was 0.805 and the Bartlett amount was equal to 792.520 (p › 0.0001), which indicated a suitability for factor analysis on variables. In this study, after removing variables extracted from flat overlapping (less than 0.5), three factors with eigenvalues greater than one, were extracted and variables were classified based on the load factor (the share of each factor in the formation and after varimax factor rotation) (**Table 2**).

**Table 2:** Special value and variance extracted from the individual obstacles and loading variables

Factor Name	Variable	Factor load
Lack of awareness and inadequate knowledge	Unfamiliarity with statistical principles	0.686
	Insufficient knowledge of research methodology	0.814
	Lack of fluency in English to use the academic resources	0.622
	Unawareness of latest research	0.780
	Inability to identify areas of research	0.801
Lack of time and business	Lack of time and business	0.868
	Family responsibilities	0.858
Lack of motivation	Lack of motivation	0.731
	No benefit for the patient and clients	0.865

The first element, called the lack of knowledge, found in an amount of 3.27 alone explained the 31.622% of the total variance. The second factor, identified as the lack of time and business, with a particular value of 1.57, explained the 18.85% of the total variance, and the third factor, known as the lack of motivation, was found with the unique value of 1.155 and described the 15.11% of the total variance. Overall, these three factors explained 65.344% of the total variance in the individual barriers section, which indicated the high value of the explained variance. **Table 3** presents the variable positioning of the different obstacles factors with loadings.

**Table 3:** Special value and variance extracted from individual obstacles and loading variables

Factor Name	Variable	Factor load
Lack of organizational support	Lack of sufficient incentives to researchers from the authorities	0.627
	Lack of adequate funding for research	0.429
	Insufficient income from research (compared to other activities)	0.739
	The lack of use from results of the study	0.502
	Lack of management points in the promotion of research	0.751
	Income inequality in the adoption and implementation of research projects	0.628
Lack of library services and advice	Lack of specialist librarian to guide the use of resources	0.730
	Weak forces in Research Consulting	0.734
	Lack of access to information resources like library	0.686
	Lack of access to information sources, such as Medline, Internet, and Journal	0.566
Lack of facilities in the sample	Problems related to sampling and Statistics	0.757
	The time limit for research	0.743
Lack of access to samples of the various research	The unavailability of a sample	0.772
	Forcing the use of a specific framework	0.705
Lack of cooperation	Lack of cooperation from health staff	0.829
	Lack of cooperation from the authorities of health centers	0.856
Lack of facilities and Consulting	Lack of facilities and equipment	0.849
	The unavailability of research consulting forces	0.677

Based on the findings in the personal barriers, the KMO (Kaiser-Meyer-Olkin Measure of Sampling Adequacy KMO) amount was 0.810 and the Bartlett amount was equal to 1254.853 (p › 0.0001), which indicated the suitability for factor analysis on variables. In this study, after removing the variables extracted after the flat overlapping (less than 0.5), six factors with eigenvalues greater than one were extracted, and variables were classified based on the load factor (the share of each factor in the formation and after varimax factor rotation).

The first element, called the lack of organizational support was found in an amount of 4.667 alone, and it explained the 25.927% of the total variance. The lack of library services, lack of facilities in the sample, lack of access to sample a variety of research, lack of cooperation, lack of resources and consulting, each explained 25.927%, 8.893%, 6.686%, 6.214%, 5.666%, of the total variance. Overall, these six factors explained 64.806% of the total variance in the organizational barriers section. 

When comparing scores of personal barriers based on the three factors extracted, there was a significant gender difference in the average scores of the individual barriers based on the three factors extracted, within the third factor (lack of motivation). Therefore, it means that the lack of motivation of male experts than of female experts was noted to be a stronger barrier (p = 0.043). The lack of time and business factor for the professionals with a history of fewer than five years was the higher factor than the one of the experts with a history of over ten years of working history (p = 0.012). The experts who working in hospitals and the people working in health centers (p = 0.006) and the college staff (p = 0.002) reported a lack of time and business factor as a larger obstacle. The study of the individual regarding education, marital status, and employment status did not show any significant differences.

The mean scores of the organizational barriers were based on six extracted factors, within the first factor (lack of corporate support). No significant difference was observed regarding the service location. In other words, experts working in health centers reported this as a stronger barrier compared with the University employees (p = 0.039). Within the second factor (Lack of library services and advice), people working in health centers in hospitals expressed a firmer barrier than the employees in hospitals (p = 0.01). In the context of the fourth factor (lack of access to samples and research various ways) for the staff in the health centers, it was a bigger issue than for the college teams (p = 0.021). For the experts who had the history of research, the lack of organizational support was more important than for people without a working record (p = 0.008). Significant differences were detected in the study of organizational factors by gender, marital status, education level and employment status.

## Discussion

The findings showed that in the area of personal obstacles, the lack of time and business factor had the highest average score. The lack of time and business was the most important factor preventing research in Sereshti, Abedini, Sharifi and Sabzevari [**3**,**5**,**12**,**13**]. Sereshti believes that one of the factors of this problem is the higher level of women as compared to men and concludes that, because of housekeeping and its responsibilities, women have less time to do the research [**3**]. About 60% of the total participants in the present study were women. Moreover, it should be noted that conducting research is one of the main responsibilities of the university professors, and it is not one of the primary functions and expected tasks from experts working in clinical practice [**12**]. In addition, performing different tasks and doing research is time-consuming.

In the area of organizational barriers, the major obstacle is the lack of motivation from the authorities. Sereshti also expressed this variable as one of three critical barriers in the organizational barriers in his study [**3**]. In general, experts work in an organization that does not expect them to do any research activities, his primary focus being clinical practice [**12**]. In addition, this could be one of the leading causes of the lack of motivation from the authorities.

Comparing the average score for individual obstacles with a mean rating of the organizational barriers, it was shown that the regulatory barriers average was higher than the personal obstacles average (p › 0.0001). In two studies conducted on teachers and staff nurses in hospitals and the University of Medical Sciences, Sereshti and his colleagues reported an average score for the organizational barriers higher than the individual obstacles [**3**,**14**] and quoted from Royal that the regulatory barriers are more significant than the environmental and personal obstacles [**3**]. In their study on University faculty members, Sabzevari and his colleagues reported that the personal barriers average is higher than the corporate ones [**13**]. It could be indicated that the working experts face more organizational obstacles than the teachers, in doing the research. 

The results of the factor analysis on the variables of individual barriers showed that by using varimax, the variables affecting the individual barriers were extracted in the form of three elements. These factors included the lack of awareness and knowledge, lack of time and lack of motivation. In this context, Harrison (2005) found that it is important to train the health care personnel factor as a leading culture factor. He added that we should accept that we cannot become experts in the field of research and development over the night, and it is better that this education was provided regionally and locally rather than in other cities [**11**]. Providing the educational opportunities so that the staff pursued a master’s degree and a doctorate in the field, improved the capacity for active research [**15**]. Dodani (2007) observed an increase in the capacity of regional research, as an unsolved challenge in developing countries [**1**]. Nchinda (2002) also wrote about this. Experience showed that in most of the developing countries there is a lack of experts in most fields of study and do enough multidisciplinary research is necessary for the experts identified and trained in a wide range of disciplines and research topics [**16**]. Salmon (2007) identified the lack of time in addition to the lack of skills, a central issue in research, in a study he performed on doctors. He performed numerous studies, which have shown the lack of time to do research. He also quoted form Marx (1997) in everyday language, the bounce to adherence, the time limits, which are culturally considered an acceptable excuse. On the other hand, in order to reduce the burden when a behavior is questioned - “there was no time” mainly means that attitude was of a lower priority than the other activities, and that it is more of an excuse rather than an explanation [**17**]. Harrison (2005) noted the lack of time in doing research as a considerable obstacle [**11**]. Salmon (2007) reported in his study that individuals see research as useless and concluded that curiosity and the sense of finding answers were low in the subjects [**17**]. Overall, Stange (1996) believed that significant barriers existed in the research in primary care, being difficulties in translating the knowledge-based clinical practice into a study with strict implementation, lack of critical scholars, weak growth and the need for a competitive research culture that the researchers face [**18**].

The results of the analysis showed that the variables of the organizational barriers were classified into six factors. These six factors included the lack of corporate support, lack of library services and advice, lack of facilities in the sample, lack of access to samples of the various research, lack of cooperation, lack of facilities and consulting. The lack of facilities and consulting explained 64.80% of the total variance. Harrison (2005) believed that the organizational barriers and the administrative barriers were the most significant obstacles, in the sense that they did not support studies because their mind was occupied with reducing the waiting list of the patients and did not see researching as a good way to spend credits, thus not showing any genuine support for doing it. The current efforts for the realization of the staff without adequate managerial and organizational support will probably not last [**11**]. Mwandumba (2009) saw financial assistance as the cornerstone of the development of research capacity and believed that the researchers needed to be trained to identify the appropriate funding sources that would enable them to compete. He also saw the role of collaboration and cooperation within and outside the organization in strengthening the research capacity in high quality through the sharing of ideas, knowledge, technologies, and research findings [**19**]. Nchinda (2002) believed that experts faced the indifference or even stubbornness of the officials regarding their work. He added that insufficient staff motivation, apart from the other co-workers, poor access to resources and low salaries were all obstacles that complexed research and prevented the timely response to the needs of the ever-changing country researchers trained [**16**].

Sereshti (2007) counted the lack of facilities, the lack of research and the lack of motivation on the advice of the researchers from the authorities as three main reasons respectively [**3**]. Sabzevari and his colleagues reported the three top obstacles as restrictive administrative regulations, lack of funding and lack of equipment. Habib wrote that there should be efforts for the materials and tools, so that the research that needed less funding and was more related to mind and brain and human abilities had the first priority [**13**]. Jafari and his colleagues counted the lack of a central database, lack of new sources of information, lack of facilities such as a library research as deterrents of research [**20**]. Lots of researchers showed that the lack of facilities and equipment is one of the important organizational obstacles in the way of doing research [**4**,**21**].

## Conclusion

Improve the capacity of experts through the proper training to do quality research, and provide corporate support - equipment, support equipment, and other support to develop the research capabilities.

**Acknowledgment**

We would like to thank the Deputy of Research and Technology of the University of Medical Sciences in the adoption and implementation of this partnership. We would also like to thank all our esteemed colleagues for the participation in the study.
